# Vitamin D status in female military personnel during combat training

**DOI:** 10.1186/1550-2783-7-38

**Published:** 2010-12-14

**Authors:** Nancy E Andersen, J Philip Karl, Sonya J Cable, Kelly W Williams, Jennifer C Rood, Andrew J Young, Harris R Lieberman, James P McClung

**Affiliations:** 1 Military Nutrition Division, US Army Research Institute of Environmental Medicine, Natick, MA, 01760, USA; 2 Experimentation and Analysis Element, Directorate of Basic Combat Training, Fort Jackson, SC, 29207, USA; 3 Pennington Biomedical Research Center, Louisiana State University System, Baton Rouge, LA, 70808, USA

## Abstract

Vitamin D is an essential nutrient for maintaining bone health. Recent data suggest that vitamin D and calcium supplementation might affect stress fracture incidence in military personnel. Although stress fracture is a health risk for military personnel during training, no study has investigated changes in vitamin D status in Soldiers during United States (US) Army basic combat training (BCT). This longitudinal study aimed to determine the effects of BCT on 25-hydroxyvitamin D (25(OH)D) and parathyroid hormone (PTH) levels in female Soldiers. Serum 25(OH)D and PTH were assessed in 74 fasted Soldier volunteers before and after an 8-week BCT course conducted between August and October in Columbia, South Carolina. In the total study population, 25(OH)D levels decreased (mean ± SD) from 72.9 ± 30.0 to 63.3 ± 19.8 nmol/L (*P *< 0.05) and PTH levels increased from 36.2 ± 15.8 to 47.5 ± 21.2 pg/mL (*P *< 0.05) during BCT. Ethnicity affected changes in vitamin D status (ethnicity-by-time interaction, *P *< 0.05); 25(OH)D decreased (*P *< 0.05) in both Hispanic and non-Hispanic whites, but did not change in non-Hispanic blacks. Ethnicity did not affect BCT-induced changes in PTH. These data indicate that vitamin D status in female Soldiers may decline during military training in the late summer and early autumn months in the Southeastern US. Future studies should strive to determine the impact of military clothing and seasonality on vitamin D status, as well as the functional impact of declining vitamin D status on bone health.

## Introduction

Vitamin D is an essential nutrient for maintaining bone health. Sufficient levels of vitamin D, assessed by measuring 25-hydroxyvitamin D (25(OH)D) concentrations, can be defined as the 25(OH)D concentration that either prevents an increase in parathyroid hormone (PTH), a serum calcium regulator suppressed by 25(OH)D, or optimizes calcium absorption [1]. Vitamin D sufficiency may prevent fractures in adults, while insufficiency may result in poor bone mineralization, pain, and rickets in children [2]. According to data collected in the third National Health and Nutrition Examination Survey (NHANES III), women aged 14-30 years in the United States (US) consume less vitamin D from dietary and supplemental sources than other age groups [3]. Suboptimal vitamin D intake and diminished vitamin D status may be particularly important during periods of intense physical activity such as military training, as compromised bone health could contribute to the development of stress fractures. Decrements in nutritional status during US Army basic combat training (BCT) have been documented in female Soldiers [4]. As over 300,000 women serve in the US military, understanding the specific nutritional needs of this population during physical training is critical.

Poor vitamin D status has been associated with an increased incidence of stress fracture in Soldiers [5]. Stress fractures are one of the most debilitating injuries in military recruits, and occur most often in military personnel beginning exercise regimens that include unaccustomed and physically-demanding activities. During military training regimens such as BCT, up to 21% of female recruits are diagnosed with at least one stress fracture [6]. The impact of stress fractures on military readiness is notable; the attrition rate of female Soldiers with diagnosed stress fractures may be up to 60% [6,7].

Exploring the effects of BCT on vitamin D status in female Soldiers may contribute to the development of improved guidance regarding sunlight exposure and dietary vitamin D intake for stress fracture prevention. The objective of this pilot study was to investigate the effects of military training on vitamin D status and PTH, an indirect vitamin D status indicator, in female military personnel [8]. Previous studies indicate differences in both stress fracture prevalence and vitamin D status between ethnicities [6,9]. Therefore, a secondary objective was to examine the relationship between vitamin D and PTH levels and ethnicity.

## Methods

Volunteers were recruited from a population of female Soldiers entering US Army BCT at Fort Jackson, Columbia, SC. This study was approved by the Human Use Review Committee at the US Army Research Institute of Environmental Medicine (USARIEM). Human volunteers participated in these studies after providing their free and informed voluntary consent. Investigators adhered to Army Regulation 70-25 and US Army Medical Research and Materiel Command Regulation 70-25 on the use of volunteers in research. The training course was conducted over an 8-week period between August and October of 2007. The data presented in this short report were collected as a subset of a previously published randomized, placebo-controlled trial designed to determine the role of iron status for maintaining health and performance during BCT [10,11]. The cohort examined in this analysis consumed placebo capsules containing cellulose each day; these volunteers were not provided with iron containing capsules nor did they have access to other dietary supplements. From the initial study [10,11], blood samples were available for the assessment of vitamin D status and PTH levels from 74 volunteers (Table 1).

**Table 1:** Volunteer demographics1

	**Pre**	**Post**
Age (yrs)	21 ± 4	
Height (cm)	162 ± 6	
Weight (kg)	62 ± 9	62 ± 7
Ethnicity (n)		
Non-Hispanic whites	39	
Non-Hispanic blacks	24	
Hispanic whites	11	

^1^Data collected during the initial (pre) and final (post) wks of basic combat training; means ± SD.

Basic combat training consists of both physical and military-specific training. The course is divided into three phases. The first phase consists of physical training and learning Army values and policies. The second phase involves weapons training and various assault courses. The final phase involves field exercises and the evaluation of skills taught during the first two phases. Physical training activities during BCT include road marching, distance running, and sprinting. Soldiers also participate in muscle strength training activities, including calisthenics, sit-ups, and push-ups. Military activities include obstacle courses, didactic classroom instruction, and standing in formation [11]. Comprehensive measures of the ambulatory activity experienced during BCT have been reported elsewhere [12]. During physical training activities, which typically occur in the early morning (0500-0700) hours, Soldiers are required to wear uniforms consisting of shorts and short-sleeved shirts. At all other times Soldiers are generally required to wear the Army Combat Uniform (ACU), which consists of boots, long pants, long-sleeved shirts, and caps. While wearing the ACU, only the hands and face are exposed to sunlight. Although the use of sun protection is recommended during BCT, data regarding the use of such products was not collected during this study.

Blood was collected from fasted Soldiers by antecubital venipuncture, processed on site, frozen, and shipped to USARIEM or the Pennington Biomedical Research Center (Baton Rouge, LA) for further analysis. Serum 25(OH)D (Immunodiagnostic Systems, Fountain Hills, AZ) and PTH (Siemens 2000, Los Angeles, CA) levels were determined using commercially available immunoassays. Self-reported ethnic characteristics were used to separate subjects into 3 groups (non-Hispanic white, n = 39; non-Hispanic black, n = 24; Hispanic white, n = 11) for statistical analysis.

Statistical analysis was performed using the Statistical Package for the Social Sciences v. 15.0 (SPSS Inc., Chicago, IL). A two-factor ANOVA with repeated measures was used to test for main effects of both ethnicity and time, as well as ethnicity-by-time interactions in 25(OH)D and PTH. When a significant ethnicity-by-time interaction was observed, post hoc analyses with Bonferroni adjustments were conducted to identify within- and between-group differences. Significance was set at *P *≤ 0.05 for all tests.

## Results

**Figure 1:**
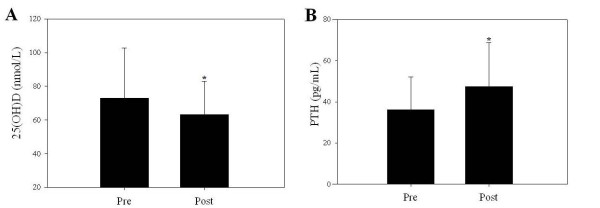
**(A) Mean serum 25-hydroxyvitamin and (B) parathyroid hormone levels in female Soldiers pre- and post-basic combat training**. Serum 25-hydroxyvitamin D, 25(OH)D; parathyroid hormone, PTH. n = 74; values are means ± SD. Asterisks (*) indicate significant differences (*P *< 0.05) from pre-values.

**Figure 2:**
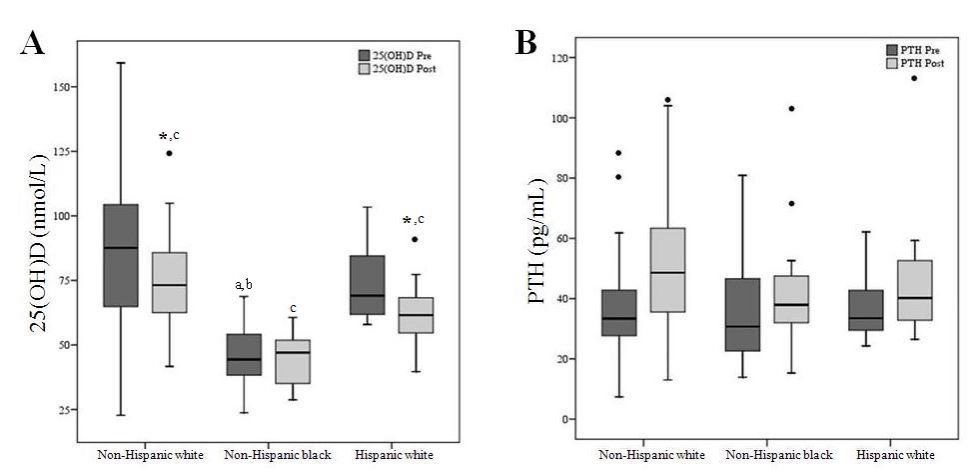
**(A) Boxplots of serum 25-hydroxyvitamin D and (B) parathyroid hormone levels in female Soldiers pre- and post-basic combat training by ethnicity**. Serum 25-hydroxyvitamin D, 25(OH)D; parathyroid hormone, PTH; basic combat training, BCT. n = 74; non-Hispanic white, n = 39; non-Hispanic black, n = 24; Hispanic white, n = 11. Boxes represent the middle 50^th ^percentile, and vertical lines extend to the 10^th ^and 90^th ^percentiles. Median values are marked by a line within each box. Values below the 10^th ^percentile or above the 90^th ^percentile are identified by solid circles (•). A two-factor repeated measures ANOVA with Bonferroni adjustments was utilized to determine the effects of time and ethnicity on 25(OH)D and PTH levels. Asterisks (*) indicate significant differences between mean values pre- and post-BCT within ethnicities (*P *< 0.05). ^a^differences between mean values of non-Hispanic whites and non-Hispanic blacks pre-BCT (*P *< 0.01); ^b^differences between mean values of non-Hispanic blacks and Hispanic whites pre-BCT (*P *< 0.05); ^c^differences between mean values of all ethnic groups post-BCT (*P *< 0.05).

Overall, mean 25(OH)D levels declined during BCT (72.9 ± 30.0 vs 63.3 ± 19.8 nmol/L, *P *< 0.05, Figure 1A). Ethnicity affected changes in vitamin D status (ethnicity-by-time interaction, *P *< 0.05); 25(OH)D decreased (*P *< 0.05) in non-Hispanic whites, and in Hispanic whites, but did not change in non-Hispanic blacks (Figure 2A). Furthermore, mean 25(OH)D levels were lowest (*P *< 0.05) in non-Hispanic blacks at both time points. In the total study population, PTH levels increased over the course of BCT (36.2 ± 15.8 vs 47.5 ± 21.2 pg/mL, *P *< 0.05, Figure 1B); however, this change was independent of ethnicity (Figure 2B).

## Discussion

Vitamin D is a critical nutrient for active populations, as it contributes to effective bone remodeling and calcium homeostasis. The major finding of this pilot study is that vitamin D status in female Soldiers declines during military training in the summer and early autumn months in the Southeastern US. This finding was unanticipated, as we expected the vitamin D status of female Soldiers to remain static or increase due to sunlight exposure during BCT, as much of the training occurs outdoors during daylight hours. Although further research is required to elucidate the mechanism, we hypothesize that the type of clothing worn during BCT, coupled with potentially inadequate dietary vitamin D intake may contribute to the observed decline in vitamin D status. Recent studies have utilized 25(OH)D values of ≤75 nmol/L as an indicator of suboptimal vitamin D status [8,13,14]. If this cutoff is applied to the data gleaned from the present study, 57% of subjects entered BCT with 25(OH)D levels <75 nmol/L, and 75% completed BCT below the cutoff value, indicating that the majority of Soldiers demonstrated suboptimal vitamin D status during BCT.

Our findings demonstrate ethnic differences in vitamin D status. Similar to previous reports, 25(OH)D levels were lowest in non-Hispanic blacks and tended to be highest in non-Hispanic whites [15-17]. Furthermore, vitamin D status declined significantly in non-Hispanic and Hispanic whites, but not in non-Hispanic blacks. We observed an increase in PTH levels within the total study population; however, PTH levels did not differ between ethnic groups. Although some studies have demonstrated higher PTH levels in blacks, this relationship appears to be inconsistent [15,17]. It is possible that physical activity associated with BCT had an interactive effect on vitamin D and PTH levels, as others have described complex relationships between physical activity, vitamin D status, PTH levels, and bone health [18,19].

To the best of our knowledge, this preliminary study is the first to describe a decline in vitamin D status in female military personnel during US Army training. Limitations of our study include a lack of data regarding the use of sun protection and the collection of data during only one cycle of BCT which occurred during the late summer and early autumn months. Future studies should aim to investigate the health and functional consequences of this decline, especially in relation to effects on bone strength and stress fracture incidence and its mechanism, as declines in vitamin D status may negatively influence calcium absorption and compromise bone health. For this reason, vitamin D and calcium supplementation may prove efficacious for preventing stress fracture during military training or other physical training regimes [20]. Dietary intake assessment may help to illustrate the nutritional factors contributing to changes in vitamin D status during training and differences between ethnic groups, and may also provide support for recommending nutrition education or intervention during BCT. Furthermore, future studies should assess the effects of military uniforms coupled with the seasonal nature of changes in vitamin D status during military training.

## Competing interests

The authors declare that they have no competing interests.

## Authors' contributions

All authors read and approved the final manuscript. NA and JK participated in data collection, statistical analysis, and manuscript preparation. SC, KW, and JR participated in data collection and study management. HL and AY contributed to study design and manuscript preparation. JM served as the principal investigator and contributed to study design, data collection, and manuscript preparation. All authors read and approved the final draft.
